# Draft Genome Sequences of *Klebsiella variicola* Plant Isolates

**DOI:** 10.1128/genomeA.01015-15

**Published:** 2015-09-10

**Authors:** Esperanza Martínez-Romero, Jesús Silva-Sanchez, Humberto Barrios, Nadia Rodríguez-Medina, Jesús Martínez-Barnetche, Juan Téllez-Sosa, Rosa Elena Gómez-Barreto, Ulises Garza-Ramos

**Affiliations:** a Centro de Ciencias Genómicas (CCG), Universidad Nacional Autónoma de México (UNAM), Cuernavaca, Morelos, México; b Instituto Nacional de Salud Pública (INSP), Centro de Investigación Sobre Enfermedades Infecciosas (CISEI), Departamento de Diagnóstico Epidemiológico, Cuernavaca, Morelos, México; c Instituto Nacional de Salud Pública (INSP), Centro de Investigación Sobre Enfermedades Infecciosas (CISEI), Cuernavaca, Morelos, México

## Abstract

Three endophytic *Klebsiella variicola* isolates—T29A, 3, and 6A2, obtained from sugar cane stem, maize shoots, and banana leaves, respectively—were used for whole-genome sequencing. Here, we report the draft genome sequences of circular chromosomes and plasmids. The genomes contain plant colonization and cellulases genes. This study will help toward understanding the genomic basis of *K. variicola* interaction with plant hosts.

## GENOME ANNOUNCEMENT

*Klebsiella variicola* comprises clinical as well as plant and insect associated bacteria (1–4). The species was described in 2004 mainly on the basis of a distinct phylogenetic position and low DNA-DNA hybridization to related species (4). Now, PCR-multiplex may be used for a quick identification of *K. variicola* (5) and a wide SNP analysis supported the taxonomic status of the species (6). *K. variicola* isolates represent a small proportion of *Klebsiella* recovered from hospitalized patients (6, 7), and thus they seem less virulent than *Klebsiella pneumoniae* (1). However, in other cases, K. variicola infections are highly lethal (7). Plant, insect and clinical *K. variicola* strains are capable of fixing nitrogen and promoting plant growth (8); they seemingly have different epidemiological dynamics in comparison to *K. pneumoniae* (9). A genomic analysis would reveal *K. variicola* virulence or antibiotic resistance genes and genes involved in its association with plants.

The endophytic isolates studied in this work were originally obtained from inside tissues from maize shoots (strain 3), banana leaves (strain 6A2), or sugar cane stem (strain T29A) (2, 4). Total genomic DNA from *K. variicola* isolates were extracted and purified using the DNeasy Kit (Qiagen, Germany). Whole-genome sequences were generated using pyrosequencing on the 454 Roche FLX Titanium (T29A and 6A2 strains) and Plus (3 strain) platform. Reads (99.9% above Q40) longer than 500 bp were used for *de novo* assembly with the CLC Genomics Workbench version 4.0 (CLC bio). The total sequence data are as follows. *K. variicola* T29A genome (227,982 reads with 30- to 943-bp length range): a total of 126 contigs, with an *N*_50_ of 97,031 bp and estimated genome size of 5,865,668 bp (including the 200-kb and 80-kb plasmids) with 18× coverage; *K. variicola* 3 genome (463,271 reads with 24- to 1,508-bp length range): a total of 24 contigs, with an *N*_50_ of 374,829 bp and estimated genome size of 5,512,858 bp with 25× coverage; and *K. variicola* 6A2 genome (205,051 reads with 30- to 943-bp length range): a total of 206 contigs, with an *N*_50_ of 49,243 bp and estimated genome size of 5,925,914 bp (including the 200-kb, 150-kb and 140-kb plasmids) with 15× coverage. Gene prediction and annotation were carried out using the bioinformatic MicroScope platform (10). In the *K. variicola* T29A, 3, and 6A2 genomes, respectively, a total of 5,751, 5,387, and 5,906 coding DNA sequences; 67, 77, and 60 tRNA genes; and 4, 6, and 2 rRNA were found. The average G+C content was similar in the three genomes (56.9 to 57.3).

 A BLAST analysis showed a *nifJ*-*nifQ* cluster (with 20 genes), *pyrG*, acetyl-CoA acetyltransferase (KPK_0844), *purF*, *argG*, *lysA*, *ivlI*, *adA*, *uvrA*, *dinF*, *fumC*, and *acrA* genes. The *K. variicola* T29A and 6A2 genomes contained the *celK* gene that encodes to cellulose 1,4-beta-cellobiosidase protein, and the *bglH* gene that encodes to Aryl-phospho-beta-d-glucosidase protein is contained in the T29A, 3, and 6A2 genomes. The sequenced genomes are similar to the previously reported *K. pneumoniae* 342 (actually *K. variicola*) genome (1). A total of 4,680 core genes were determined among the *K. variicola* genomes included in this study.

### Nucleotide sequence accession numbers. 

The annotated genomes sequences are available at the European Nucleotide Archive under accession numbers CXPA01000001 (*K. variicola* T29A), CXOY01000001 (*K. variicola* 3), and CXPC01000001 (*K. variicola* 6A2).
